# Diet quality and nutritional risk in patients with Wilson’s disease: a cross-sectional study

**DOI:** 10.3389/fnut.2025.1643645

**Published:** 2025-09-11

**Authors:** Shou-hong Lin, Mei-ling Yang, Yi Zeng, Ting-Ting Wu

**Affiliations:** ^1^Department of Neurology, The First Affiliated Hospital, Fujian Medical University, Fuzhou, China; ^2^Department of Nursing, The First Affiliated Hospital, Fujian Medical University, Fuzhou, China

**Keywords:** Wilson disease, dietary quality index-international, mini nutritional assessment, diet quality, nutritional risk

## Abstract

**Objective:**

This study aimed to evaluate diet quality in patients with Wilson’s disease (WD), identify associated factors, and investigate the relationship between diet quality and nutritional risk in this population.

**Methods:**

This cross-sectional study enrolled patients with WD at a tertiary hospital in Fujian Province from November 2023 to February 2025. Dietary quality was evaluated using the Dietary Quality Index-International (DQI-I), and nutritional risk was assessed with the Mini Nutritional Assessment (MNA). A DQI-I score of 61 (median) was used to dichotomize dietary quality. Logistic regression identified factors associated with lower DQI-I scores. Associations between DQI-I and nutritional risk were examined using three models: unadjusted (Model 1); adjusted for age, sex, and BMI (Model 2); and further adjusted for educational attainment, disease duration, smoking, clinical classification, comorbidities, chelator, zinc preparation, self-reported medication adherence, liver damage, 24-h urinary copper, and albumin (Model 3). A linear regression scatterplot was used to visualize the association.

**Results:**

A total of 91 patients with WD were included, with a mean DQI-I score of 59.51 ± 7.42. Overall, 74.7% were either malnourished or at risk of malnutrition. Lower DQI-I scores were significantly associated with female sex, lower educational attainment, longer disease duration, and smoking (all *p* < 0.05). In the unadjusted model (Model 1), a DQI-I score ≤61 was associated with a 34.83-fold increased risk of malnutrition and a 4.98-fold increased risk of nutritional risk (both *p* < 0.05), compared to scores >61. These associations remained significant after multivariable adjustment (Models 2 and 3). DQI-I scores were moderately correlated with nutritional risk (*r* = 0.448, *p* < 0.001).

**Conclusion:**

Patients with WD exhibit suboptimal dietary quality and a high prevalence of nutritional risk. Lower DQI-I scores independently predict malnutrition, emphasizing the utility of diet quality assessment in clinical care. Early identification of at-risk individuals, coupled with targeted, dietitian-led interventions, may improve dietary variety, mitigate nutritional risk, and support better long-term outcomes.

## 1 Introduction

Wilson disease (WD), also known as Hepatolenticular Degeneration (HLD), is an autosomal recessive disorder affecting copper metabolism, with clinical onset ranging from infancy to late adulthood (8 months to 74 years) (1, 2). Its prevalence is estimated at 5.87 per 100,000 in China (3), and 2 per 100,000 in the UK (4). Research indicates that the 10-year cumulative mortality rate for patients with WD is 9.3% (including both outpatient and inpatient cases) (5). WD is phenotypically heterogeneous, typically presenting with hepatic and/or neurological manifestations (4), and is influenced by genetic, environmental, and dietary factors (6). Once diagnosis, patients require lifelong copper-chelation therapy and dietary copper restriction to mitigate copper accumulation and slow disease progression (7).

Nutritional management in WD is particularly challenging. Copper-restricted diets necessitate the exclusion of shellfish, organ meats, nuts, legumes, and whole grains, all of which are also rich sources of protein, fiber, and vitamins (8). This restriction compromises nutrient density and dietary diversity. To compensate for energy needs, patients may turn to high-fat diets, leading to excessive fat intake and metabolic stress, which could influence disease phenotype and progression (8–10). Chelation therapy additionally disrupts the metabolism of key micronutrients (e.g., zinc, calcium, iron, phosphorus) (11). Zinc supplementation is closely associated with gastrointestinal symptoms (12). In patients with WD, the incidence of gastritis may reach 65.2%, and duodenal ulcers are also common (12), further compromising nutrient absorption (13). WD’s clinical heterogeneity compounds nutritional risk. Cirrhosis is present at diagnosis in 25–54% of cases (4), and chronic liver disease increases protein catabolism and reduces synthesis, exacerbating malnutrition (14). Other common features such as olfactory dysfunction (15) and sleep disturbances (16) may reduce appetite and caloric intake (17, 18). Despite the central role of diet in WD management, data on dietary quality and nutritional status in this population remain scarce.

To date, only limited studies have explored nutritional differences across clinical phenotypes in WD (19), and few have investigated dietary factors contributing to malnutrition. No study has systematically evaluated overall dietary quality or its association with nutritional risk in adults with WD. The Dietary Quality Index-International (DQI-I), a validated tool assessing dietary variety, moderation, adequacy, and balance (20), has been linked to metabolic syndrome, cardiovascular disease, and diabetes in diverse populations (21–23). Similarly, the Mini Nutritional Assessment (MNA) is widely used to identify malnutrition risk and predict adverse outcomes (24, 25). Applying both DQI-I and MNA in WD may reveal diet-related risk patterns and clarify their association with nutritional status, addressing a critical gap in current research.

This study aimed to: (1) assess dietary quality among adults with WD; (2) identify factors associated with poor dietary quality; and (3) evaluate the association between DQI-I scores and nutritional risk. Findings may support evidence-based dietary interventions and inform more precise, standardized nutritional care in WD management.

## 2 Methods

### 2.1 Participants

This cross-sectional study enrolled patients with WD from the rare disease outpatient clinic of a tertiary hospital in Fujian Province between November 2023 and February 2025. Inclusion criteria were: (1) confirmed diagnosis of WD based on Leipzig scoring system (≥4 points; 8th International Meeting on Wilson’s Disease, 2001) (26) or ATP7B gene mutation testing; (2) Age ≥18 years; (3) Disease duration >1 year with clinical stability. Exclusion criteria were: (1) requirement for enteral nutrition due to dysphagia; (2) cognitive or communication impairments interfering with participation; (3) refusal to participate; (4) severe psychiatric or psychological disorders; (5) advanced cardiac, pulmonary, hepatic, or renal insufficiency, severe neurological symptoms, or malignancy.

### 2.2 Measurements

Patient data were collected using a structured questionnaire encompassing the following domains:

#### 2.2.1 General information

(1) Demographics: age, sex, marital status, living conditions, income, education attainment, and healthcare coverage.
(2) Health Behaviors and Lifestyle: Dietary information sources included self-directed learning (via multimedia, magazines, books, and experiences shared by peers) and professional guidance obtained during outpatient visits, through educational brochures, or inpatient counseling. Additional factors assessed were nutritionist consultations, smoking status, alcohol consumption, and occupational physical activity. Work intensity was categorized as light (≥75% sedentary), moderate (25–75% sedentary), or heavy (≤40% sedentary) based on Chinese labor classification standards (27). Exercise frequency and duration were also recorded.
(3) Anthropometry: Weight and height were measured using a calibrated ultrasonic device (Melien MSG003S, Shenzhen, China). BMI was calculated as weight (kg)/height^2^ (m^2^).

#### 2.2.2 Disease-related information

a. Disease status includes clinical classification, manifestations, disease progression, and comorbidities. Clinical classification consists of the following subtypes (6): (1) Hepatic subtype: patients with overt liver symptoms such as jaundice, anorexia, nausea, coagulopathy, and ascites; (2) Neurological subtype: patients exhibiting neurological features, including movement disorders, tremors, gait abnormalities, dysphagia, speech difficulties, sialorrhea, and psychiatric disorders, with or without liver involvement (28); (3) Asymptomatic subtype: patients identified during routine physical examinations; (4) Others: a minority of patients presenting with joint pain, arthritis, or renal symptoms at disease onset; b. Laboratory parameters, such as albumin, total bilirubin, INR, creatinine, ALT, and AST; c. Copper metabolism indicators, including serum ceruloplasmin, 24-h urinary copper, urinary copper, and serum copper levels; d. Medication use, encompassing chelators and zinc supplements, along with self-assessment of adherence; e. Additional information: The Activities of Daily Living (ADL) score evaluates patients’ self-care capabilities, whereas the Child-Pugh score determines the severity of liver cirrhosis (29).

#### 2.2.3 Food frequency questionnaire

Dietary intake over the past year was assessed using a validated 110-item FFQ adapted for Chinese populations (30). The FFQ demonstrated acceptable test–retest reliability and validity, with median values of 0.53 and 0.46, respectively. Food categories included grains, dairy, meats, eggs, fish, tubers, vegetables, legumes, pickled foods, fruits, and snacks. Frequency responses ranged from “never” to “≥2 times/day.” Nutrient intake was estimated using the Chinese Food Composition Table (6th edition) (31) and used to calculate DQI-I scores.

#### 2.2.4 Dietary quality index-international

The DQI-I comprises four domains (20): variety (0–20), adequacy (0–40), moderation (0–30), and overall balance (0–10). Variety evaluates intake diversity across and within food groups; adequacy assesses essential nutrients (e.g., fiber, protein, iron, calcium, vitamin C); moderation penalizes excessive intake of fat, cholesterol, sodium, and energy-dense foods; and balance reflects macronutrient distribution and fatty acid ratios. Total scores range from 0 to 100, with higher scores indicating better dietary quality.

#### 2.2.5 Mini nutritional assessment

The 18-item MNA evaluates four domains: anthropometry (BMI, weight loss, arm/calf circumference), general health (mobility, medication, depressive symptoms), dietary intake (meal frequency, fluid/food consumption), and self-perceived nutritional status. A score of 24–30 indicates normal nutritional status; 17–23.5, nutritional risk; and <17, malnutrition. The scale has a high sensitivity of up to 96% for assessing nutritional risk (24).

### 2.3 Data collection

Face-to-face interviews were conducted by trained researchers to obtain demographic data, administer the FFQ and MNA, and clarify responses. For participants with limited literacy, item-by-item explanations and visual aids (e.g., food photographs) were provided to enhance response accuracy. Clinical classification, symptom profiles, laboratory values, copper metabolism indices, and ADL scores were extracted from medical records. Liver injury severity was determined by abdominal ultrasound performed by two independent specialists (mild to moderate liver damage is defined as cases that do not fulfill the criteria for normal liver findings or cirrhosis). Child-Pugh scores were determined by a designated team of hepatology physicians.

### 2.4 Statistics

All analyses were performed using SAS version 9.4 (SAS Institute, Cary, NC, United States). Categorical variables were summarized as counts and percentages [n (%)], with between-group comparisons assessed using chi-square or Fisher’s exact tests. Normality of continuous variables was evaluated using the Shapiro–Wilk test and histogram inspection. Normally distributed variables were presented as means ± standard deviations and compared using t-tests or ANOVA.

To identify factors associated with low dietary quality, DQI-I scores were dichotomized at the median (≤61 vs. > 61), and logistic regression was used. Associations between DQI-I and nutritional risk (MNA categories) were explored using three logistic models: Model 1 (unadjusted); Model 2 (adjusted for age, sex, and BMI); and Model 3 (further adjusted for educational level, disease duration, smoking, clinical classification, comorbidities, chelator, zinc preparation, self-reported medication adherence, liver damage, 24-h urinary copper, and albumin). To further assess the linear relationship, a scatter plot with linear regression was constructed between DQI-I and MNA scores. A two-tailed *p*-value <0.05 was considered statistically significant.

### 2.5 Quality control

Standardized protocols for patient recruitment, data collection, and assessment were uniformly applied across all study procedures. Inclusion and exclusion criteria were strictly enforced to reduce heterogeneity. Dietary intake data were verified against the 6th edition of the Chinese Food Composition Table (31) by two registered dietitians. Data were independently entered by two researchers and validated by a third for consistency. All personnel involved underwent standardized training, with periodic audits and quality checks to ensure protocol adherence and data reliability. Questionnaires were excluded if incomplete or completed in ≤5 min.

## 3 Results

### 3.1 Patient characteristics

Among the 95 individuals initially recruited, 91 were included in the final analysis after excluding 4 participants with incomplete questionnaires, yielding a completion rate of 95.8%. The cohort comprised 44 males (48.4%) and 47 females (51.6%), with a mean age of 34.7 years (SD 11.8) and a mean BMI of 21.6 (SD 2.9). Most participants (91.2%) reported adherence to a copper-restricted diet; however, only 2.2% had ever received dietary counseling from a nutritionist. Regarding disease duration following diagnosis, 32.9% had been diagnosed for ≤5 years, 30.8% for >5 to 10 years, and 36.3% for >10 years. Hepatic involvement was the predominant clinical subtype (65.9%), followed by neurological manifestations (29.7%). Evidence of liver injury was present in 74.7% of participants, with 22.0% classified as having mild to moderate damage and 52.8% diagnosed with cirrhosis. Among those with cirrhosis, 91.7% were classified as Child-Pugh class A, indicating compensated liver function, as seen in Table 1.

**Table 1:** Baseline characteristics and determinants of DQI-I scores.

Variable	n (%)	DQI**-**I ≤ 61 (*n* = 48)	DQI**-**I > 61 (*n* = 43)	Statistic	*P-value*
Sex					
Male	44(48.35)	17(35.42)	27(62.79)	*χ^2^* = 6.806	**0.009**
Female	47(51.65)	31(64.58)	16(37.21)		
Age(*x̅* ± *s*), years	34.7 ± 11.8	33.6 ± 11.6	36.0 ± 12.0	*t* = 0.961	0.339†
Educational attainment					
Elementary school	36(39.56)	24(50.00)	12(27.91)	*χ^2^* = 8.569	**0.014**
Middle or high school	33(36.26)	18(37.50)	15(34.88)		
College degree or above	22(24.18)	6(12.50)	16(37.21)		
Income, yuan					
<4,000	54(59.34)	28(58.33)	26(60.47)	*χ^2^* = 0.043	0.836
≥4,000	37(40.66)	20(41.67)	17(39.53)		
Marital status					
Married	51(56.04)	25(52.08)	26(60.47)	*χ^2^* = 0.647	0.421
Other	40(43.96)	23(47.92)	17(39.53)		
Number of siblings					
Only child	6(6.59)	4(8.33)	2(4.65)	–	0.063*
1 sibling	34(37.36)	14(29.17)	20(46.51)		
2 siblings	24(26.37)	17(35.42)	7(16.28)		
3 siblings	11(12.09)	3(6.25)	8(18.60)		
4 or more siblings	16(17.58)	10(20.83)	6(13.95)		
Living conditions					
Living with family	64(70.33)	31(64.58)	33(76.74)	*χ^2^* = 1.607	0.205
Living alone	27(29.67)	17(35.42)	10(23.26)		
Work intensity					
Light	54(59.34)	26(54.17)	28(65.12)	–	0.388*
Moderate	29(31.87)	16(33.33)	13(30.23)		
Heavy	8(8.79)	6(12.50)	2(4.65)		
Disease duration, years					
<5	30(32.97)	10(20.83)	20(46.51)	*χ^2^* = 6.819	**0.033**
5–10	28(30.77)	17(35.42)	11(25.58)		
>10	33(36.26)	21(43.75)	12(27.91)		
Treatment methods					
Medication only	8(8.79)	5(10.42)	3(6.98)	–	0.891*
Diet control + medication	79(86.81)	41(85.42)	38(88.37)		
Dietary control (prescribed but not used)	4(4.40)	2(4.17)	2(4.65)		
Healthcare coverage					
Self-pay	24(26.37)	11(22.92)	13(30.23)	*χ^2^* = 2.029	0.363
Medical insurance	23(25.27)	15(31.25)	8(18.60)		
Commercial insurance	44(48.35)	22(45.83)	22(51.16)		
Dietary sources					
Home-cooked meals	68(74.73)	34(70.83)	34(79.07)	*χ^2^* = 0.815	0.367
Order takeout or dine out	23(25.27)	14(29.17)	9(20.93)		
Sources of nutrition education					
Self-study	44(48.35)	27(56.25)	17(39.53)	*χ^2^* = 2.538	0.111
Healthcare professionals	47(51.65)	21(43.75)	26(60.47)		
Nutritionist consult					
No	89(97.80)	47(97.92)	42(97.67)	–	1.000*
Yes	2(2.20)	1(2.08)	1(2.33)		
Smoking					
No	53(58.24)	23(47.92)	30(69.77)	*χ^2^* = 4.453	**0.035**
Yes	38(41.76)	25(52.08)	13(30.23)		
Alcohol use					
No	6(6.59)	4(8.33)	2(4.65)	*χ^2^* = 0.080	0.777
Yes	85(93.41)	44(91.67)	41(95.35)		
Exercise frequency					
No exercise	37(40.66)	15(31.25)	22(51.16)	–	0.051*
1–2 times/week	22(24.18)	17(35.42)	5(11.63)		
3–4 times/week	9(9.89)	4(8.33)	5(11.63)		
>4 times/week	23(25.27)	12(25.00)	11(25.58)		
Exercise duration					
Less than 30 min	37(40.66)	19(39.58)	18(41.86)	*χ^2^* = 0.224	0.894
30–60 min	34(37.36)	19(39.58)	15(34.88)		
More than 1 h	20(21.98)	10(20.83)	10(23.26)		
BMI, kg/m^2^	21.6 ± 2.9	21.9 ± 2.8	21.3 ± 2.9	*t* = 0.883	0.380†
Chelator					
No	5(5.49)	2(4.17)	3(6.98)	*χ^2^* = 0.016	0.899
Yes	86(94.51)	46(95.83)	40(93.02)		
Zinc preparation					
No	4(4.40)	2(4.17)	2(4.65)	*χ^2^* = 0.000	1.000
Yes	87(95.60)	46(95.83)	41(95.35)		
Medication adherence					
Rarely remembers	4(4.40)	2(4.17)	2(4.65)	–	0.772*
More than half the time	4(4.40)	2(4.17)	2(4.65)		
Usually remembers	31(34.07)	14(29.17)	17(39.53)		
Regularly every day	52(57.14)	30(62.50)	22(51.16)		
ADL, points	100.0(95.0,100.0)	100.0(95.0,100.0)	100.0(95.0,100.0)	*Z* = −0.447	0.655#
Comorbidities					
No	84(92.31)	45(93.75)	39(90.70)	*χ^2^* = 0.023	0.88
Yes	7(7.69)	3(6.25)	4(9.30)		
Clinical classification					
Hepatic subtype	60(65.93)	33(68.75)	27(62.79)	–	0.203*
Neurological subtype	27(29.67)	15(31.25)	12(27.91)		
Asymptomatic type	2(2.20)	0(0.00)	2(4.65)		
Others	2(2.20)	0(0.00)	2(4.65)		
Clinical phenotype					
Liver damage					
No liver damage	23(25.27)	10(20.83)	13(30.23)	*χ^2^* = 3.327	0.19
Mild to moderate liver damage	20(21.98)	14(29.17)	6(13.95)		
Liver cirrhosis	48(52.75)	24(50.00)	24(55.81)		
Dystonia	50(54.95)	23(47.92)	27(62.79)	*χ^2^* = 2.027	0.155
Tremor	39(42.86)	20(41.67)	19(44.19)	*χ^2^* = 0.059	0.808
Limb rigidity	43(47.25)	21(43.75)	22(51.16)	*χ^2^* = 0.500	0.479
Behavioral abnormalities	1(1.10)	0(0.00)	1(2.33)	–	0.473*
Kayser-Fleischer ring	59(64.84)	27(56.25)	32(74.42)	*χ^2^* = 3.284	0.07
Others	9(9.89)	4(8.33)	5(11.63)	*χ^2^* = 0.030	0.862
Urinary copper, μmol/L	0.65(0.25, 1.34)	0.66(0.25, 1.59)	0.65(0.25, 1.15)	*Z* = −0.060	0.952#
24-h urinary copper, μg/24 h	83.4(36.2, 191.5)	85.4(33.3, 180.1)	79.9(40.8, 221.1)	*Z* = 0.052	0.958#
Ceruloplasmin, mg/L	30.0(20.0, 60.0)	37.8(30.0, 60.0)	30.0(20.0, 60.0)	*Z* = −1.258	0.208#
Serum copper, μmol/L	2.71(1.66, 3.86)	2.92(2.00, 4.07)	2.55(1.55, 3.50)	*Z* = −1.248	0.211#
Albumin, g/L	42.9 ± 5.0	43.9 ± 4.6	41.8 ± 5.3	*t* = 1.985	0.051†
INR	1.09(1.04, 1.18)	1.11(1.05, 1.19)	1.09(1.02, 1.15)	*Z* = −1.082	0.279#
Creatinine, μmol/L	65.0(56.0, 93.0)	65.0(56.5, 94.0)	66.0(56.0, 86.0)	*Z* = −0.239	0.811#
Total bilirubin, μmol/L	12.9(10.2, 17.8)	12.9(10.4, 16.4)	12.9(9.5, 19.0)	*Z* = 0.195	0.845#
ALT, U/L	30.0(20.0, 42.0)	31.0(20.5, 43.0)	30.0(18.0, 42.0)	*Z* = −0.605	0.545#
AST, U/L	27.0(21.0, 34.0)	27.5(20.5, 37.5)	27.0(22.0, 34.0)	*Z* = −0.127	0.898#

“*” indicates the use of Fisher’s exact test; “†” indicates the use of the t-test. “#” indicate the use of rank-sum test. Items with statistically significant values are indicated in bold.

### 3.2 Dietary quality and its determinants

The mean DQI-I score was 59.51 ± 7.42, with subdomain means as follows: variety 15.61 ± 3.42, adequacy 28.21 ± 6.21, moderation 13.42 ± 5.11, and balance 2.36 ± 3.11. Based on the median cutoff (61), participants were grouped into DQI-I ≤ 61 (*n* = 48, 52.75%) and DQI-I > 61 (*n* = 43, 47.25%). Significant differences were observed across sex, education, disease duration, and smoking status. The DQI-I ≤ 61 group had a higher proportion of females (64.58% vs. 37.21%), lower education levels (12.5% vs. 37.21%), longer disease duration (>10 years: 43.75% vs. 27.91%), and more smokers (52.08% vs. 30.23%) (all *p* < 0.050), as shown in Table 2.

**Table 2:** Distribution of DQI-I scores and components across nutritional risk categories.

		Grouping				
Variable	Total (*n* = 91) (x̅ ± s)	Malnutrition (*n* = 25) (x̅ ± s)	Nutritional risk (*n* = 43) (x̅ ± s)	Well-nourished (*n* = 23)	Statistic	*P-value*
Variety	15.61 ± 3.42	13.82 ± 3.51	15.62 ± 3.31	17.61 ± 2.42	*F* = 8.415	**<0.001‡**
Overall variety	12.32 ± 2.21	11.43 ± 2.11	12.31 ± 2.21	13.22 ± 2.01	*F* = 4.277	**0.016‡**
Protein variety	3.34 ± 1.80	2.44 ± 2.06	3.30 ± 1.71	4.39 ± 0.94	*F* = 8.223	**<0.001‡**
Adequacy	28.21 ± 6.21	24.82 ± 5.61	27.82 ± 5.91	32.51 ± 4.92	*F* = 11.532	**<0.001‡**
Vegetable	4.02 ± 1.52	3.68 ± 1.86	3.88 ± 1.53	4.65 ± 0.78	*F* = 2.905	0.060‡
Fruit	2.09 ± 1.44	1.72 ± 0.98	1.95 ± 1.36	2.74 ± 1.82	*F* = 3.531	**0.033‡**
Cereal	4.32 ± 1.00	3.88 ± 1.01	4.44 ± 0.91	4.57 ± 1.04	*F* = 3.642	**0.030‡**
Fiber	1.80 ± 1.17	1.36 ± 0.86	1.65 ± 1.04	2.57 ± 1.34	*F* = 8.213	**<0.001‡**
Protein	4.45 ± 1.16	3.48 ± 1.66	4.77 ± 0.65	4.91 ± 0.42	*F* = 16.442	**<0.001‡**
Iron	3.42 ± 1.57	2.92 ± 1.82	3.37 ± 1.51	4.04 ± 1.19	*F* = 3.252	**0.043‡**
Calcium	4.20 ± 1.22	4.28 ± 1.14	3.98 ± 1.26	4.52 ± 1.20	*F* = 1.588	0.210‡
Vitamin C	3.87 ± 1.62	3.52 ± 1.83	3.74 ± 1.62	4.48 ± 1.24	*F* = 2.405	0.096‡
Moderation	13.42 ± 5.11	15.22 ± 5.53	13.51 ± 4.81	11.32 ± 4.62	*F* = 3.549	**0.032‡**
Total fat	1.43 ± 2.16	2.32 ± 2.61	1.19 ± 2.09	0.91 ± 1.41	*F* = 3.218	**0.044‡**
Empty foods	3.82 ± 2.37	3.12 ± 2.52	3.63 ± 2.50	4.96 ± 1.46	*F* = 4.150	**0.018‡**
Saturated fat	1.52 ± 2.34	2.52 ± 2.69	1.47 ± 2.30	0.52 ± 1.47	*F* = 4.754	**0.010‡**
Cholesterol	1.55 ± 2.29	2.28 ± 2.34	1.67 ± 2.48	0.52 ± 1.47	*F* = 3.864	**0.024‡**
Sodium	5.08 ± 1.98	4.92 ± 2.27	5.51 ± 1.30	4.43 ± 2.54	*F* = 2.385	0.098‡
Overall balance	2.36 ± 3.11	1.20 ± 2.18	2.67 ± 3.15	3.04 ± 3.61	*F* = 2.615	0.079‡
Macronutrients ratio	1.154 ± 2.12	0.60 ± 1.66	1.16 ± 2.14	1.74 ± 2.44	*F* = 1.762	0.178‡
Fatty acid ratio	1.21 ± 2.15	0.60 ± 1.66	1.51 ± 2.32	1.31 ± 2.24	*F* = 1.463	0.237‡
DQI-I score	59.51 ± 7.42	55.01 ± 6.93	59.52 ± 7.21	64.43 ± 5.12	*F* = 11.998	**<0.001‡**
DQI-I≤61[n(%)]	48(52.75)	22(88.00)	22(51.16)	4(17.39)	*χ^2^* = 24.044	**<0.001**
DQI-I>61[n(%)]	43(47.25)	3(12.00)	21(48.84)	19(82.61)		

“‡” uses F-test. Items with statistically significant values are indicated in bold.

### 3.3 Association between DQI-I and nutritional risk

According to the MNA, 27.5% of patients were malnourished, 47.3% at risk, and 25.2% well-nourished. Overall, 74.7% showed some degree of nutritional compromise. DQI-I scores differed significantly across nutritional categories, particularly in components such as diversity (total and protein), adequacy (fruit, grain, fiber, protein, iron), and moderation (total fat, saturated fat, cholesterol, and empty foods) (*p* < 0.05). Among participants with DQI-I ≤ 61, malnutrition was more prevalent and well-nourished less common (88.00% vs. 51.16% vs. 17.39%; χ^2^ = 24.044, *p* < 0.001, Table 2).

In logistic regression analysis, participants with DQI-I ≤ 61 had a 34.83-fold increased odds of malnutrition and a 4.98-fold higher odds of being at nutritional risk compared to those with DQI-I > 61(Model 1). These associations remained significant after adjusting for age, sex, and BMI (Model 2) and further for educational attainment, disease duration, smoking, clinical classification, comorbidities, chelator, zinc preparation, self-reported medication adherence, liver damage, 24-h urinary copper, and albumin (Model 3), as depicted in Table 3.

**Table 3:** Adjusted association between DQI-I score and nutritional risk.

	Malnutrition **vs** well-nourished		Nutritional risk **vs** well-nourished	
Variable	*OR*(95%*CI*)	*P-value*	*OR*(95%*CI*)	*P-value*
Model 1				
DQI-I score				
>61	1.00(ref)		1.00(ref)	
≤61	34.83(6.908 ~ 175.65)	**<0.001**	4.98(1.450 ~ 17.074)	**0.011**
Model 2				
DQI-I score				
>61	1.00(ref)		1.00(ref)	
≤61	36.92(6.753 ~ 201.81)	**<0.001**	6.17(1.635 ~ 23.277)	**0.007**
Model 3				
DQI-I score				
>61	1.00(ref)		1.00(ref)	
≤61	45.55(5.520 ~ 375.89)	**<0.001**	5.14(0.868 ~ 30.421)	0.071

Items with statistically significant values are indicated in bold.

**Figure 1:**
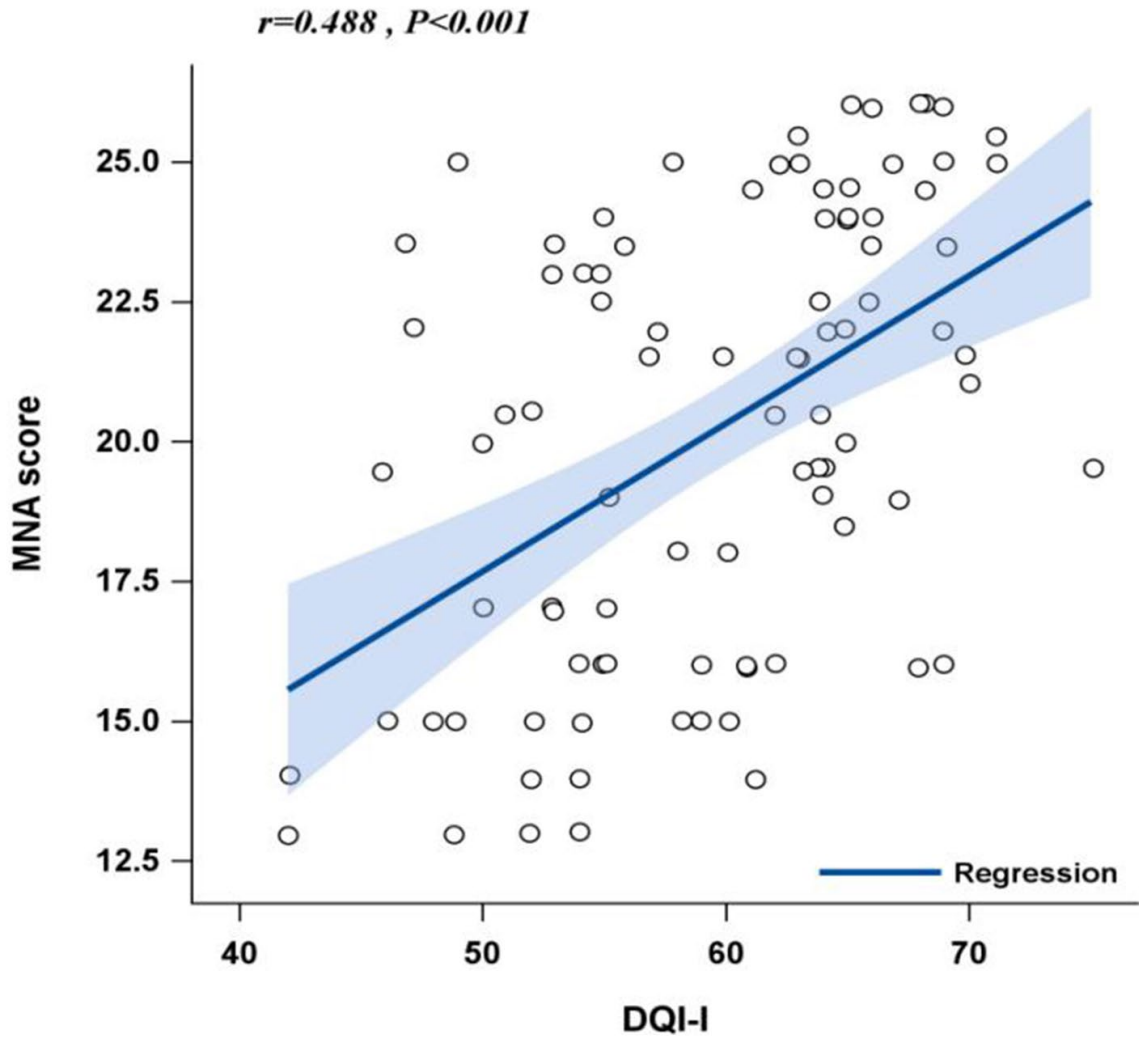
Scattter plot of linear fit regression for DQI-I and MNA scores.

Correlation analysis demonstrated a moderate positive relationship between DQI-I and MNA scores (*r* = 0.448, *p* < 0.001). Linear regression confirmed this trend, with higher DQI-I scores predicting improved nutritional status (Figure 1).

## 4 Discussion

To our knowledge, this study is the first to apply both the DQI-I and MNA to evaluate dietary quality and nutritional risk in WD patients. The findings reveal suboptimal dietary quality and a high prevalence of nutritional risk (74.7%), suggesting a dual burden of diet-related imbalance and malnutrition. Low DQI-I scores were significantly associated with female sex, lower educational attainment, smoking, and longer disease duration. Multivariate analyses confirmed that lower DQI-I scores independently predicted higher malnutrition risk, with odds ratios exceeding 30-fold. These results indicate that dietary quality plays a crucial role in nutritional status and highlight the need for targeted dietary interventions in WD care.

In comparison to prior studies, the dietary quality of WD patients in our cohort is significantly poorer. The mean DQI-I score was 59.51 ± 7.42, which falls below the 61.3 reported in a Hong Kong adult population, even after deducting six points related to moderation of empty-calorie foods (32). Higher scores have been consistently observed in older Chinese populations, 64.4 ± 9.6 (33), 64.5 ± 9.5 (34), 64.27 ± 9.6 (35), and 65.1 ± 9.2 (36), all exceeding those observed in the WD cohort. Compared to individuals with type 2 diabetes of similar age and BMI, WD patients exhibited reduced dietary diversity (15.61 ± 3.42 vs. 18.6 ± 1.5) and elevated moderation scores (13.42 ± 5.11 vs. 9.3 ± 5.0) (37). Even when excluding empty-calorie foods, the moderation score in the WD group remained elevated relative to the diabetic cohort. This dietary pattern may reflect a prolonged avoidance of copper-rich foods, such as nuts, mushrooms, whole grains, liver, and shellfish. However, this pattern may inadvertently promote excessive intake of animal fats, which are low in copper but high in saturated fat and cholesterol (11). Such imbalances are detrimental and may exacerbate hepatic injury; animal studies have shown that high-fat, high-calorie diets exacerbate hepatic injury in WD (10), and excess saturated fat is associated with adverse lipid profiles and increased risk of neurodegeneration (38). These findings highlight the need to improve dietary Variety while moderating harmful fat intake to optimize overall nutritional quality in WD.

Sociodemographic factors significantly influenced dietary patterns. Female patients, due to lower recommended daily copper intake (1.3 mg/day vs. 1.6 mg/day in males) (39), may follow stricter copper restriction, particularly in the context of hepatic presentations which are more common in women (40). Greater treatment adherence observed among female patients (40) may paradoxically contribute to reduced dietary diversity. Lower educational attainment and longer disease duration were also associated with lower dietary quality, potentially due to limited nutritional literacy (41) and entrenched restrictive dietary behaviors (39). Furthermore, smoking was inversely associated with DQI-I scores, consistent with prior literature linking smoking to poor dietary habits and increased risk of nutrient deficiencies (42, 43). Interestingly, BMI was not associated with dietary quality, aligning with previous findings that DQI-I does not reliably correlate with adiposity (44). This suggests that BMI may not be a valid proxy for dietary quality in WD patients, particularly in the context of disease-driven metabolic adaptations. Additionally, this study identified no significant correlations between clinical phenotype, manifestations, comorbidities, copper metabolism parameters, standard nutritional indices, or medication use and DQI-I or nutritional risk. These findings emphasize the necessity of incorporating individualized social and behavioral factors in the nutritional management of mild WD, rather than focusing exclusively on pathological indicators.

To our knowledge, this is the first study to assess nutritional risk in WD patients using the MNA scale. Our research found a malnutrition prevalence of 74.7% among WD patients, markedly higher than the 43.41% reported in a Chinese cohort of WD patients using the NRS2002 tool (19). Both studies demonstrated comparable BMI values (21.6 ± 2.9 vs. 22.78 ± 4.21 kg/m^2^), suggesting that the observed difference may be attributable to the greater sensitivity of the MNA in detecting nutritional deficits (45). A DQI-I score ≤ 61 was associated with a 34.83-fold higher odds of malnutrition (Model 1). The results remained robust even after adjusting for confounding factors, including BMI, clinical classification, liver impairment, comorbidities, and zinc supplementation, in Models 2 and 3, underscoring its strong predictive utility.

Among DQI-I components, lower scores in dietary diversity, adequacy, and moderation were independently associated with malnutrition, highlighting the central role of diet quality. Unlike findings in metabolic dysfunction-associated steatotic liver disease (MASLD), where adequacy and moderation were predominant (46), our results suggest that dietary diversity is particularly critical in WD—likely due to prolonged avoidance of copper-rich foods. Although international guidelines advise limiting liver, shellfish, mushrooms, legumes, dried fruits, whole grains, and chocolate, most of these pose negligible copper risk unless consumed in large quantities (11). Excessively restrictive copper-limited diets may therefore reduce dietary diversity and micronutrient intake, thereby increasing nutritional risk. Higher adequacy scores were associated with lower risk, while higher moderation scores—driven by saturated fat, total fat, and cholesterol—were linked to increased risk. Interestingly, greater intake of empty foods (refined carbohydrates and fats) was inversely associated with malnutrition, possibly reflecting compensatory energy intake in undernourished individuals. However, given the established U-shaped relationship between carbohydrate intake and mortality (47), overcompensation may pose additional metabolic risks. These findings support the need for nuanced dietary guidance in WD—balancing copper restriction with improved dietary diversity and adequacy, while avoiding excessive fat intake. Dietitian-led interventions may mitigate nutritional risk and improve long-term outcomes in this vulnerable population.

Despite the importance of nutrition in WD, a substantial gap in professional guidance remains. Although 91.2% of patients reported adherence to a copper-limited diet, only 2.2% had received dietary counseling from a registered dietitian. This disconnect between dietary restriction and professional support highlights a major barrier to optimal nutritional care. International guidelines recommend that professional guidance from dietitians can help avoid overly restrictive copper diets or unnecessary anxiety related to dietary issues (48), yet real-world integration of dietitians into routine WD care is limited. There is an urgent need for a structured, dietitian-led approach that integrates dietary guidance and optimal meal timing adjustments—given that 94.5% of patients use both chelators and zinc—across inpatient, outpatient, and follow-up settings. This strategy aims to enhance DQI-I scores and mitigate nutritional risks in this vulnerable population.

This study has several limitations. First, the cross-sectional design restricts causal inferences regarding the relationship between dietary quality and nutritional outcomes. Second, the exclusion of minors may limit the generalizability of the findings across the entire age spectrum of WD. Third, dietary intake was assessed using a FFQ developed for the general Chinese population, which may be susceptible to recall bias and may not adequately reflect regional dietary variations. Fourth, the exclusion of daily caloric intake assessment limited our ability to fully encompass key dimensions of dietary nutrition, potentially affecting the accurate evaluation of patients’ overall nutritional status. Fifth, although the MNA has not been specifically validated in WD populations, it was selected for its comprehensive approach to evaluating nutritional status. Sixth, the severity of liver injury was determined based on abdominal ultrasound findings, without the use of liver elastography for more precise assessment. Lastly, the absence of standardized classifications for the DQI-I related to low dietary quality prompted this study to adopt a median-based stratification approach, informed by prior research. However, this may have affected precision due to the limited sample size. Future studies should seek to enhance sample size and incorporate a healthy control group, employing quartile divisions to refine the identification of low dietary quality. Furthermore, investigating the significance of genotype differences in dietary nutrients is warranted. Large-scale multicenter longitudinal studies are crucial for validating these findings and facilitating targeted dietary interventions.

## 5 Conclusion

This study identified poor diet quality and high nutritional risk among adult WD patients. Key determinants of low DQI-I included female sex, lower educational attainment, longer disease duration, and smoking. A DQI-I score ≤61 was strongly associated with malnutrition. Targeted interventions to dietary diversity—while moderating fat intake—may help mitigate nutritional risk and improve long-term outcomes in this population. Integration of registered dietitians into routine WD management is recommended.

## Data Availability

The raw data supporting the conclusions of this article will be made available by the authors, without undue reservation.
